# Transcriptomic and physiological analysis identifies a gene network module highly associated with brassinosteroid regulation in hybrid sweetgum tissues differing in the capability of somatic embryogenesis

**DOI:** 10.1093/hr/uhab047

**Published:** 2022-01-05

**Authors:** Ruirui Zhao, Shuaizheng Qi, Ying Cui, Ying Gao, Shuaifei Jiang, Jian Zhao, Jinfeng Zhang, Lisheng Kong

**Affiliations:** 1 National Engineering Laboratory for Tree Breeding, Key Laboratory of Genetics and Breeding in Forest Trees and Ornamental Plants, Ministry of Education, The Tree and Ornamental Plant Breeding and Biotechnology Laboratory of National Forestry and Grassland Administration, College of Biological Sciences and Biotechnology, Beijing Forestry University, Beijing 100083, China; 2 Centre for Forest Biology, Department of Biology, University of Victoria, 3800 Finnerty Rd, Victoria, BC V8W 3N5, Canada

**Keywords:** All-BR-Regulated Genes, brassinosteroids, Horticultural plant reproductive biology

## Abstract

Somatic embryogenesis is a preferred method for large-scale production of forest trees due to its high propagation efficiency. In this study, hybrid sweetgum leaves with phase changes from mature to embryogenic state were selected as experimental material to study somatic embryo initiation. Embryogenicity ranged from high to low, *i.e.* from 45%, 25%, and 12.5% to 0, with the samples of embryogenic callus (EC), whiten leaf edge (WLI), whiten leaf (WLII), and green leaf (GL) respectively. High correlations existed between embryogenicity and endogenous brassinosteroids (BRs) (r = 0.95, *p* < 0.05). Similarly, concentrations of endogenous BRs of the sample set correlated positively (r = 0.93, 0.99, 0.87, 0.99, 0.96 respectively, *P* < 0.05) to expression of somatic embryo (SE)-related genes, *i.e. BBM*, *LEC2, ABI3, PLT2,* and *WOX2.* Hierarchical cluster and weighted gene coexpression network analysis identified modules of coexpressed genes and network in 4820 differentially expressed genes (DEGs) from All-BR-Regulated Genes (ABRG). Moreover, exogenously-supplemented epiBR, together with 2,4-D and 6-BA, increased embryogenicity of GL-sourced callus, and expression of SE- and auxin-related genes, while brassinazole (BRZ), a BR biosynthesis inhibitor, reduced embryogenicity. Evidences obtained in this study revealed that BRs involved in phase change of leaf explants and may function in regulating gene expression and enhancing auxin effects. This study successfully established protocols for inducing somatic embryogenesis from leaf explants in hybrid sweetgum, which could facilitate the propagation process greatly, and provide theoretical basis for manipulating SE competence of explants in ornamental woody plants.

## Introduction

Somatic embryogenesis (SEis) is a process during which somatic cells form the structures similar to zygotic embryos without fertilization. As an ideal means, SEis has been used for efficient plant propagation and genetic transformation. It is also used, as a model system, for studies of reproductive biology and physiology in higher plants [1]. The process of SEis involves integration of endogenous signals and gene reprogramming; thereby it is important to unlock the signals that initiate the process of embryogenesis. Several genes have been postulated involving in the acquisition of somatic embryogenic competence, which include *BABY BOOM (BBM)* [2], *LEAFY COTYLEDON (LEC)* [3], *SOMATIC EMBRYOGENESIS RECEPTOR LIKE KINASE1 (SERK1)* [4], *ABA INSENSITIVE 3 (ABI3)* [5], *AGAMOUS-LIKE (AGL)* [6], *FUSCA3 (FUS3)*, *PLETHORA (PLT)*, *WUSCHEL (WUS)* [7, 8], and etc. Ectopic expression of some genes, such as *LEC2*, *BBM*, and *WUS,* enable somatic cells to maintain their embryonic characteristics in *Arabidopsis* [2, 3, 7]. Compared with *Arabidopsis*, only a small number of SEis-related genes have been identified in forest trees and the regulatory mechanisms of these genes request further study.

Hybrid sweetgum (*Liquidambar styraciflua* × *L. formosana*) is a valuable ornamental species in horticultures due to its remarkable red leaves in the fall. It is also an economically important species for production of timber and bio fuel due to its fast growth [9]. Currently, SEis could be induced from explants of staminate inflorescence and immature zygotic embryos in sweetgum (*L. styraciflua*) [10] and hybrid sweetgum [9]. According to the theory of cell pluripotency, any differentiated, alive plant cells could dedifferentiate returning to embryonic state in suitable conditions [11]. Actually, in most cases, somatic embryos (SEs) were induced from explants of less differentiated tissues like immature zygotic embryos and male gametophytes rather than mature tissue like leaf [12]. Induction of SEis from mature tissue is related closely to explant phase change, or rejuvenation [15]. However, except inflorescence, no success has been achieved previously in inducing SE from other mature tissues in hybrid sweetgum, although the leaf is accessible easily from the plant at any ages [9, 13, 14].

Brassinosteroids (BRs) are a class of plant steroid hormones playing pivotal roles in plant growth and development, such as hypocotyl and stem elongation, cell expansion, root development, vascular differentiation, and so on [16–18]. Studies also showed that the supplementation of BRs, at the suitable concentrations, enhanced SEis in plumule [19]*, Coffea arabica* [20]*,* cauliflower [21]*,* lettuce [22] and cotton [23]. Many major BR signaling components and the framework of BR signaling pathway have been identified in *Arabidopsis* [24]. Moreover, BRs have synergistic and interdependent relationships with auxin in a wide range of developmental processes. They affect auxin flow and distribution by regulating gene expression of auxin exporters, like *PIN4* and *PIN7,* and an enzyme coding gene, *CYP79B2,* which could convert tryptophan to indole-3-acetaldoxime [25, 26]. Expression of auxin responsive genes, such as *IAA5*, *IAA19*, *IAA17,* were induced by exogenously applied brassinolide (BL), while down-regulated in the BR biosynthetic mutant *de-etilated2* (*det2*) [27, 28]. As a key transcription factor in BR signaling pathway, BZR1 could interact with antifreeze proteins (AFP) to target multiple auxin signaling factors and genes, including *AUX/IAA*, *PINs*, *TIR1* and *ARF* [29]. Nevertheless, there were few studies on the mechanisms underlying BR regulation during SEis, especially with different explants, the initial plant materials of SEis.

Aims of this research were to find key factors related to the phase change of leaf explants and SE initiation in hybrid sweetgum. We first found a special phenomenon of phase change of hybrid sweetgum leaves during *in vitro* cultures. Four types of explants were then selected and used for analyzing their embryogenicity, endogenous hormones, expression of selected genes, and etc. On the analytical results, exogenous BR and brassinazole (BRZ) were used to confirm its functions during SE induction. This research provides new insight into the mechanism underlying SEis from mature tissues. It could also benefit further studies and application practice in BR-induced SEis in woody plants.

## Results

### Explant selection, embryogenicity test and gene expression analysis

**Figure 1:**
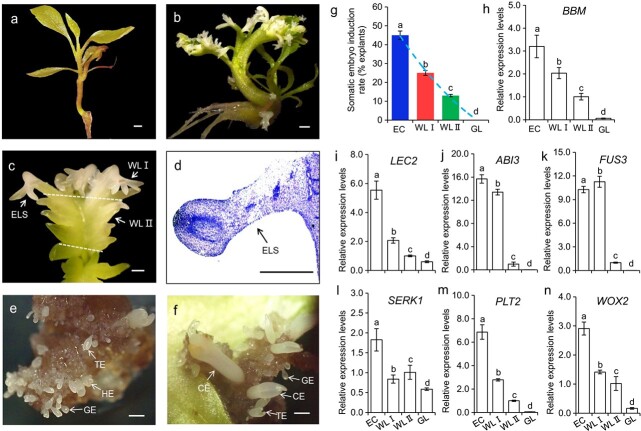
**Somatic embryogenesis system, embryogenicity test and marker gene expression.** (**a-c**) Morphological characteristics of normal (**a**) and albino (**b-c**) plantlets. WLI, white leaf I; WLII, white leaf II; ELS, embryo-like structure. Bar = 0.5 cm. (**d**) Histochemical staining of the ELS. Bar = 0.5 mm. (**e-f)** Somatic embryo induction with WLI (**e**) and WLII (**f**). GE, globular embryo; HE, heart-shaped embryos; TE, torpedo-shaped embryo; CE, cotyledonary embryo. (**g**) Somatic embryo induction rate. (**h-n**) Relative expression of marker genes. EC, embryogenic callus; GL, green leaf. Means ± SD, n = 3. Values with different letters were significantly different at *P* < 0.05.

The normal leaves of somatic seedlings were green (Fig. 1a). When SE plantlets were growing in the culture bottles in two months, some leaves were turning into white, from the tip to the base (Fig. 1b, c). A few embryo-like structures (ELS) could be observed at the leaf tip (Fig. 1c). Histological analysis revealed that the cells located at the apical area of an ELS arranged more compactly and orderly than the surrounding cells. These cells were small with thick cytoplasm. The ratio of nucleus and nucleolus to the cell volume was high (Fig. 1d), indicating that these cells could be meristematic. On the test of embryogenicity, SE formation rates using explants of embryogenic callus (EC), white part (WLI), and middle white part (WLII) were 45%, 25%, and 12.5%, respectively, while explants of normal green leaf (GL) failed to generate SE (Fig. 1e-g). As shown in Fig. 1h-n, *BBM*, *ABI3*, *FUS3, PLT2* were not expressed in samples of GL, while *LEC2*, *SERK1* and *WOX2* expressed a little in GL. The genes *BBM*, *LEC2, ABI3*, *PLT2* and *WOX2* had the highest expression levels in EC and a relatively lower expression in WLI. These genes were also expressed in WLII. The expression of *FUS3* in WLI was higher than that in EC.

### Concentrations of endogenous BRs in the explants correlated to their embryogenicity

**Figure 2:**
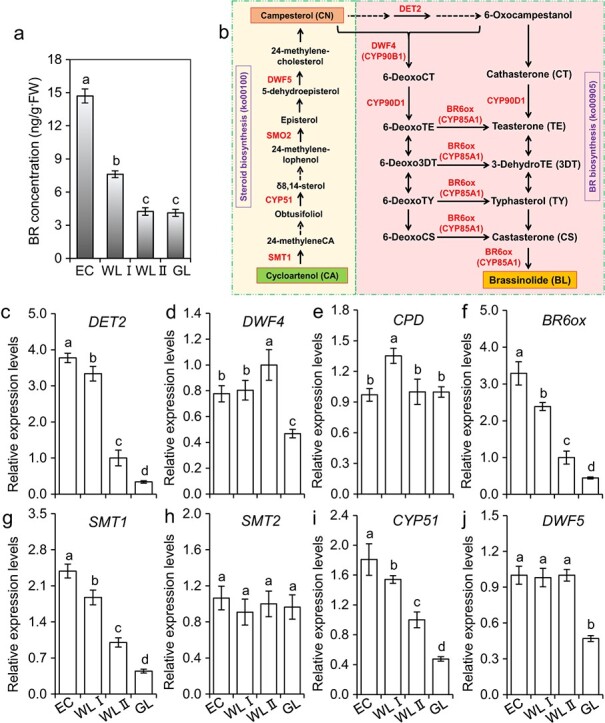
**Concentrations of endogenous BRs and the expression of genes related to BR and sterols biosynthesis.** (**a**) BR concentrations. (**b**) BR and sterols biosynthetic pathways. (**c-j**) Gene expressions. Mean ± SD, n = 3. Values with different letters were significantly different at *P* < 0.05. EC, embryogenic callus; WLI, white leaf I; WLII, white leaf II; GL, green leaf.

Endogenous BRs, abscisic acid (ABA), indole-3-acetic acid (IAA), gibberellic acid (GA_3_), gibberellin A4 (GA_4_), zeatin riboside (ZR), dihydrozeatin riboside (dhZR), indolepropionic acid (iPA), and jasmonic acid methyl ester (JA-Me) were quantified (Fig. S1). Compared with those of WLII and GL, concentrations of many plant hormones, except BRs, were very low in samples of EC and WLI (Fig. S1). Interestingly, with the increase in concentrations of endogenous BRs, the embryogenicity of different explants was also higher. The high correlation (r = 0.95, *p* < 0.05) between BRs and embryogenicity suggested an important role of BRs in SEis. The highest concentration of BRs was found in samples of EC at 14.70 ng g^−1^ FW, which was 1.93, 3.47 and 3.58 folds of those in the other three explants (Fig. 2a), whereas the highest concentrations of ABA, GA_4_, and ZR were found in GL, and these were lower in WLII, and bottomed in WLI and/or EC. Concentrations of IAA, GA_3_ and JA were relatively high in WLII and GL, lower in EC and WLI. Concentrations of dhZR and iPA were higher in GL and WLII than those in WLI and EC (Fig. S1). The transcripts of *DET2*, *BR6ox, SMT1,* and *CYP51* ranged from high to low in the samples of EC, WLI, WLII and GL. The expression levels of *DWF4*, *SMO2*, *DWF5* in EC and WLI were significantly higher than those in WLII and GL (Fig. 2b-j, *P* < 0.05). Furthermore, concentrations of endogenous BRs correlated positively to the expression of SE-specific genes, *i.e. BBM*, *LEC2, ABI3, PLT2,* and *WOX2,* r = 0.93, 0.99, 0.87, 0.99, 0.96 respectively and *P* < 0.05 (Fig. 1h-n).

### Global transcriptome changes in different explants

Full-length transcriptome was used to profile genome-wide gene expression and transcriptome changes in the explants of different embryogenicity levels. A total of 12 RNA-seq libraries were established, and high-quality sequencing was performed. An overview of the sequencing reads from 12 libraries was shown in Table S2. A total of 201.74 Gb clean data were generated, with the lowest 15.60 GB clean data in all samples and an average GC content of 43.20%. The proportion of clean reads mapped to the *L. formosana* genome ranged from 66.36 to 69.99%, and that of the uniquely mapped reads ranged from 74.74 to 68.48%. Pearson correlation analysis (Fig. S2a) and a box plot of all the samples (Fig. S2b) suggested high repeatability of the sequencing results. Based on the alignment results, 30 965 expressed unigenes were obtained and 14 197 unigenes were differentially expressed (false discovery rate (FDR) < 0.05) between at least two different samples. The number of DEGs is shown in Fig. S2c and Table S3. The highest number of DEGs of 10 610 unigenes was found in EC vs. GL, while the fewest DEGs of 1968 were found in EC vs. WLI (Fig. S2c, Table S3).

### Expression of all-BR-regulated genes (ABRG) in the explants of different embryogenicity

**Figure 3:**
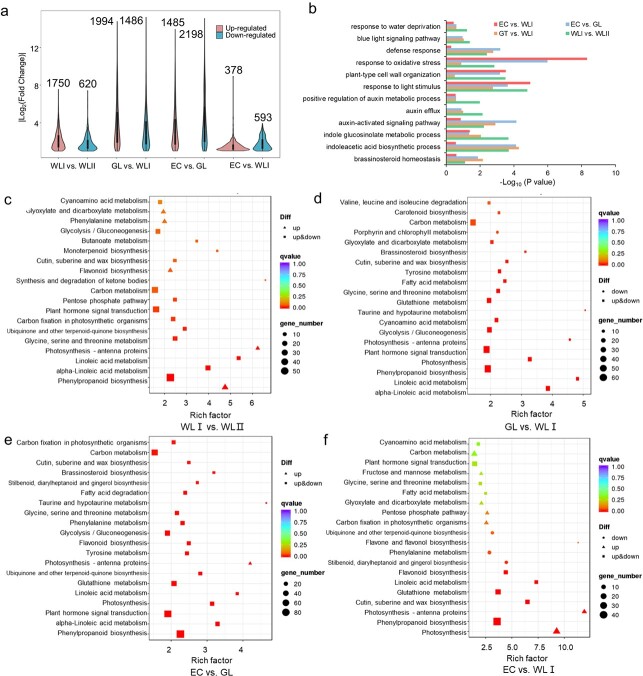
**GO and KEGG enrichment of differentially expressed ABRGs.** (**a**) The numbers and distributions of gene expression. (**b**) GO enrichment. The numbers in bar plots represent -log_10_ (FDR). (**c-f**) The top 20 KEGG pathway terms for the commonly differentially expressed ABRG genes. The numbers in bar plots represent rich factor. EC, embryogenic callus; WLI, white leaf I; WLII, white leaf II; GL, green leaf. The numbers in bar plots represent the fold enrichment compared with all DEGs.

**Figure 4:**
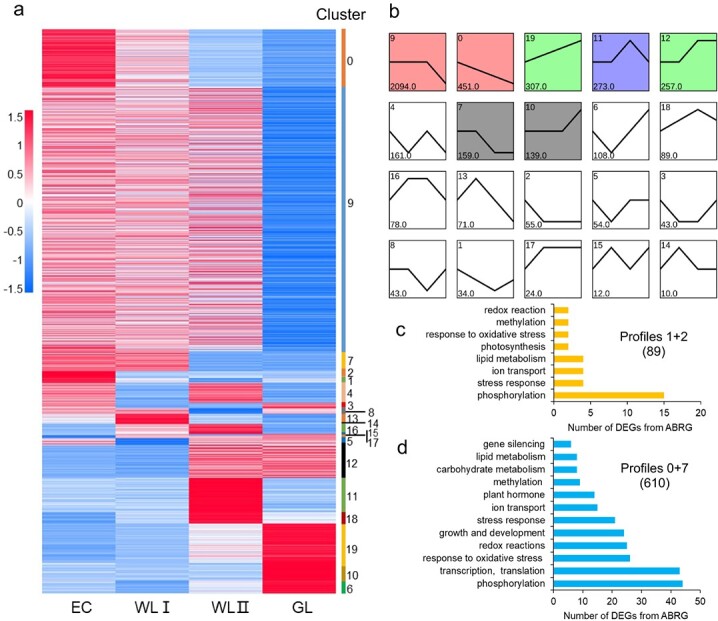
**Hierarchical cluster analysis of differentially expressed ABRGs.** (**a**) Heatmap of 4980 ABRGs. Expression levels were measured as RPKM from normalized values. Red and blue represent pairwise distances among transcripts above or below, respectively. (**b**) Magnified regions of 20 subclusters in all DEGs, black lines indicate a “consensus” of all the DEGs within each subcluster. The subclusters with significant *p*-values were colored. (**c-d**) GO classification of profiles 1 + 2 (**c**), and profiles 0 + 7 (**d**). The numbers in bar plots represent DEGs numbers. EC, embryogenic callus; WLI, white leaf I; WLII, white leaf II; GL, green leaf.

According to the 6951 All-BR-Regulated Genes (ABRG) in *Arabidopsis thaliana,* which were provided by Chen et al. [30], 7992 homology ABRG genes were obtained in hybrid sweetgum, of which 4980 ABRG genes were DEGs with FDR < 0.05 (Fig. 3a). The number of up- and down-regulated DEGs was shown in Fig. 4a. Gene Ontology (GO) and Kyoto Encyclopedia of Genes and Genomes (KEGG) enrichment analyses for pairwise comparisons were conducted (Fig. 3b-f, Fig. S3, Tab. S4). Interestingly, a large number of auxin-related genes were identified in the DEGs (Fig. 3b). The top 20 enriched KEGG pathways were shown in Fig. 3c-f. Notably, “BR biosynthesis” was enriched in EC vs. GL, and GL vs. WLI (Fig. 3d, e).

Hierarchical cluster analysis of ABRGs was shown in Fig. 4a. The clustering analysis suggested that WLI and WLII demonstrated similar expression profiles, and GL showed the greatest difference to other explants (Fig. 4a). The cluster also revealed seven main profiles (Fig. 4b). Of the 20 clustering profiles, profiles 1 + 2 (89 ABRGs) represented genes that were specifically highly expressed in EC, including genes involved in phosphorylation, stress response, ion transport, lipid metabolism, photosynthesis, oxidative stress response, methylation, and redox reaction (Fig. 4a-c). The profiles 0 + 7 (610 ABRGs) represented ABRGs specifically highly expressed in EC and WLI (Fig. 4d), corresponding to genes involved in phosphorylation, transcription and translation, response to oxidative and other stresses, redox reactions, growth and development, and so on (Fig. 4d). Brassinosteroids and auxin have synergistic and interdependent relationships in a wide range of developmental processes [17–20].

**Figure 5:**
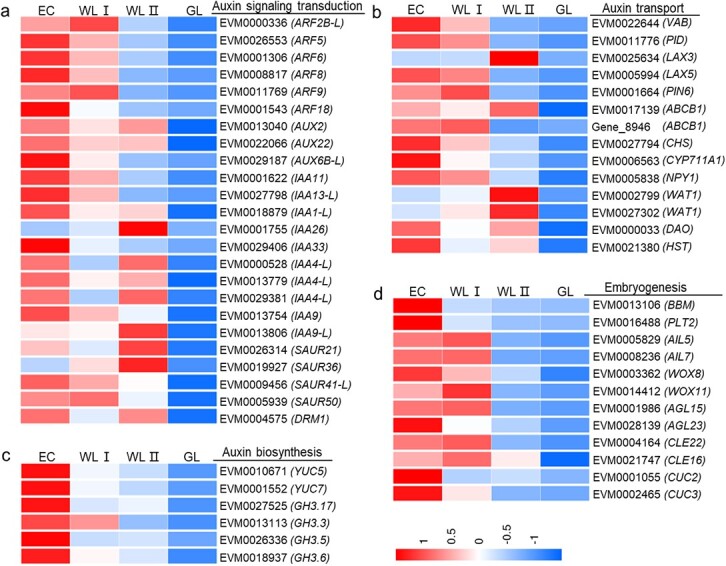
**Gene expression profile of differentially expressed ABRGs.** (**a-d**) Heatmaps showing expression of genes participated in auxin signaling (**a**), auxin biosynthesis (**b**), auxin transport (**c**), and embryogenesis (**d**). The scale represents FPKM values after z-score. FPKM, fragments per kilobase of exon model per million reads mapped. EC, embryogenic callus; WLI, white leaf I; WLII, white leaf II; GL, green leaf.

Most of the selected auxin-related and SE-related genes were significantly down-regulated with lower embryogenicity (Fig. 5a-d, Tab. S5). Genes participate in auxin signaling transduction, auxin biosynthesis, and auxin transport were included. As the DEGs were selected from profiles 1 + 2, 0 + 7 and profile 9 (Fig. 4), most of the genes have a decreased gradually expression pattern from EC, WLI, WLII to GL. A lot of regulatory genes for SEis were identified in ABRG (Fig. 5d). *AGL15* was mainly expressed in WLI and EC, but not expressed in GL. *WOX8* was expressed in all four kinds of tissues, showing a similar declining pattern. *WOX11* was specifically expressed in EC, WLI, and WL II, and the highest expression was found in WLI. *AILs* belongs to AP2-like ethylene-responsive transcription factor. *AIL5* and *AIL7* were specifically expressed in EC and WL tissues and had the highest level of expression in WLI. *CUC3* was specifically expressed in EC and WLI, with the highest expression level in EC, while *CUC2* was specifically expressed in EC. *CLAVATA3/ESR (CLE)-related protein 16* (*CLE16*) had the same expression pattern, with the expression decreased from WLI, EC, to WLII, and had no expression in GL.

**Figure 6:**
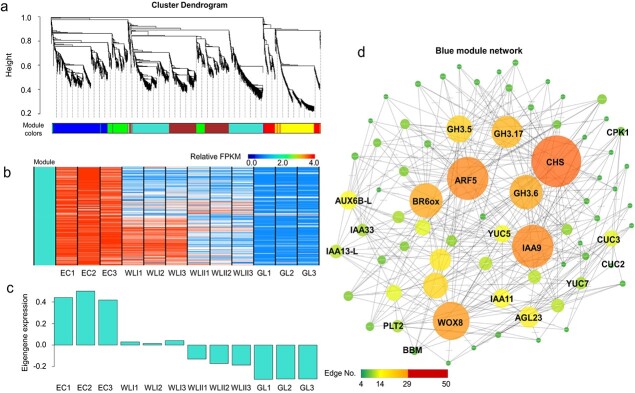
**Turquoise module genes and networks.** (**a**) Hierarchical cluster tree showing coexpression modules identified by WGCNA. Each branch in the tree is one gene. (**b**) Heat map showing the relative FPKM of each gene from turquoise module. (**c**) Eigengene expression profile for the turquoise module in different tissues. Y axis indicates the module eigengene value; X axis indicates samples. (**d**) The correlation network of turquoise module. One hundred eleven genes with the edge weight higher than 0.75 are visualized by Cytoscape.

In order to capture key changes in the gene network of different embryogenic explants, weighted gene coexpression network analysis (WGCNA) was used to analyze the co-expression relationship among 4820 DEGs of ABRGs. After filtering, genes were divided into 6 modules, which correlate with a specific tissue cluster of genes (Fig. 6a). The turquoise module represents 542 ABRGs that showed high-expression specifically in EC and WLI, and gradually decreases in WLII and GL (Fig. 6b, c). This is consistent with hierarchical cluster analysis, where 699 ABRGs in profiles 0 + 7 and profiles 1 + 2 represented highly expressed in EC and WLI were found (Fig. 4d). WGCNA can also be used to construct gene networks. In turquoise module network, network including auxin and embryogenesis related genes and genes involved BR biosynthesis was constructed (Fig. 6d).

### Effects of exogenously-applied epiBR on initiation of somatic embryogenesis with callus derived from green leaf

**Figure 7:**
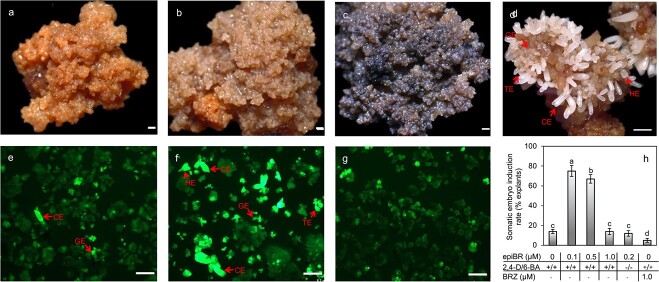
**Effects of exogenous epiBR treatment on induction of somatic embryos.** Callus treated with control (**a**), 0.1 μM epiBR (**b**) and 1.0 μM BRZ (**c**) for 30 days. (**d**) Somatic embryo induction with 0.1 μM epiBR for 90 days. Representative fluorescein diacetate (FDA)-stained callus from control (**e**), 0.1 μM epiBR (**f**) and 1.0 μM BRZ (**g**) treatments for 30 days and somatic embryo induction for 45 days. (**h**) Somatic embryo induction with different concentrations of epiBR, 2,4-D/6-BA, and BRZ. GE, globular embryo; HE, heart-shaped embryo; TE, torpedo-shaped embryo; CE, cotyledonary embryo. Mean ± SD, n = 3.Values followed by different letters were significantly different at *P* < 0.05. Bars = 0.5 mm.

Significantly higher SE induction rate was obtained with tissue treated with 0.1 and 0.5 μM epiBR than that with 0 and 1.0 μM epiBR (Fig. 7h) (*P* < 0.05). Brassinazole (BRZ) of 1.0 μM decreased the SE induction (Fig. 7h). Callus surface with 0.1 or 0.5 μM epiBR treatment improved the browning of the callus (Fig. 7a), while the callus treated with control, and BRZ browned when the tissue was cultured on SE induction medium for 30 days (Fig. 7b, c). We assessed of the viability of SE induction using FDA staining (Fig. 7e-g). After SE induction, a large number of somatic embryos appeared in tissues treated with 0.1 μM epiBR for 45 days (Fig. 7f). These SEs were at various developmental stages, *i.e.* they are globular embryos, heart-shaped embryos, torpedo-shaped embryos and cotyledonary embryos (Fig. 7f). For control, we observed very few SE induction, however, we hardly detect SE induction in 1.0 μM BRZ treatment (Fig. 7e, g). After 90 days on SE induction medium II, most somatic embryos matured with two well-developed cotyledons (Fig. 7d).

### Effect of exogenous epiBR on gene expression

**Figure 8:**
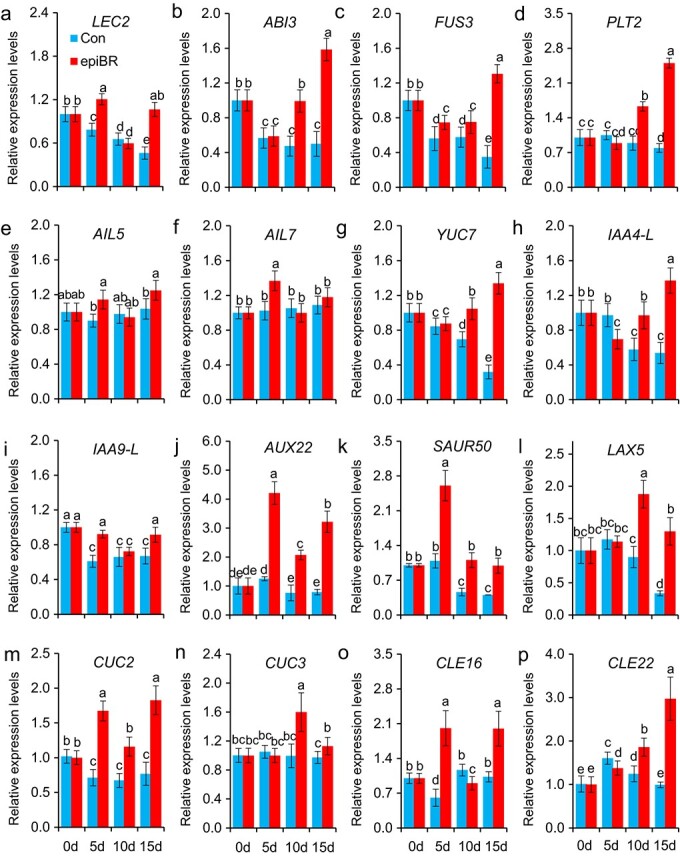
**Quantitative real-time PCR analysis of gene expression after epiBR treatments.** Mean ± SD, n = 3.Values followed by different letters were significantly different at *P* < 0.05.

After exogenous epiBR treatment, the expression of genes in Fig. 7 was detected using qRT-PCR. Many genes decreased in the control from d0 to d15, such as *LEC2, ABI3, FUS3,* and *YUC7* (Fig. 8a-c, g). However, exogenously-supplemented epiBR increased gene expression significantly (*P* < 0.05), which included *LEC2* at d5 and d15; *FUS3, AUX22, SAUR50, CUC2* at d5, d10, and d15; *ABI3*, *PLT2*, *YUC7*, *IAA4-L*, *LAX5, CUC3, CLE22* at d10 and d15; *CUC3* at d5 and d15 (Fig. 8b-p). EpiBR treatment increased *AUX22* expression to 3.4, 2.7 and 4.1 folds at d5, d10 and d15, respectively (Fig. 8j). The expression of genes related to BR and sterols biosynthesis was detected after epiBR treatment. As shown in Fig. S4, epiBR increased the expression of *DET2* at d10 and d15, *BR6ox* at d5, and *SMT1* at d5 and d15 compared with control.

## Discussion

It is one of the most interesting findings in this study that concentrations of endogenous BRs correlated positively to embryogenicity, or phase changes of explants. Also, endogenous BRs correlated positively to expression of SE-specific genes, like *BBM*, *LEC2, ABI3, PLT2,* and *WOX2*. Among these genes, over expression of *BBM1* [33], *WUX* [34]*, LEC2* [35]*, FUS3* [36]*, AIL5, AIL7* and *PLT2* genes [37] could enhance SEis in *A. thaliana*. Although, it is well known that plant hormones, especially auxin, cytokinins and GAs, participate in the process of cell rejuvenation [32]. There is little information about endogenous BRs that are linked to phase change of plant mature tissues. In this study, the increased expression of key genes participating in BR and sterols biosynthesis further proved the positive correlation of endogenous concentrations of BRs and the phase change of leaf explants. For *Liquidambar*, endogenous BRs could be used as an embryogenicity marker for explant selection. The effects of exogenously applied epiBR on SEis were significant and concentration-dependent, which was similar as the previously reported [25–28, 38]. Furthermore, treatment with BRZ, a BR biosynthesis inhibitor, reduced the SE induction rate. The increased SEis induction with exogenously applied epiBR and the induced genes may be attributed to the regulation of BRs in cell pattern regulation and specific gene expression [38].

This is the first report of inducing SEis from leaf-derived callus in *Liquidambar* spp. Explants of the whiten leaf, WLI and WLII, could produce SEs after the first round of SE induction treatment, whereas the green leaf (GL) produced SEs only after several sub-cultures and SE induction treatments. Manipulation of explants has been used extensively in different propagation methods, such as root cutting, grafting and micro propagation [15]. The purpose of manipulation is to rejuvenate the explants so that the plant material can respond to the treatment properly and result in what the needed [31]. For SEis, the suitable explants, such as immature embryos or meristems, were usually selected instead of obtaining through manipulation. With the information generated in this research, manipulating explant condition by a serial of sub-cultures and BR treatment could have large potential to increase embryogenicity of leaf explants in *Liquidambar*. This experiment is on-going in our lab with more genotypes, especially the reluctant ones in SEis.

Our transcriptomic evidences confirmed phase changes, or rejuvenation, indeed occurred in leaf explants of morphological changes in hybrid sweetgum. Several SE-related genes expressed from low to high in the explants changed from green to white in color. In *Arabidopsis,* the ELS structure often appears in the abnormal expression of certain key SE-related genes, such as the over-expressed *WUS* and the ectopic postembryonic expression of *LEC1*^32^. In our study, high expression of *BBM, LEC2, ABI3*, *PLT2* and *WOX2* could be the major cause for phase change and ELS formation. Results of the embryogenicity test could also reflect the phase change directly and obviously. It is the common knowledge that the best plant materials for initiating SEis should be of the least differentiated state, or embryogenic. The explants of EC of the highest SE induction rate, or embryogenicity was embryogenic. Explants of WLI were rejuvenated the most completely in the leaf explants, whereas the green leaf demonstrated no embryogenicity. During SE-induction treatment the GL explant produced no embryognic callus. After *in vitro* sub-cultures for several times, the GL-sourced callus could produce a few SE, which indicates further rejuvenation may have occurred during *in vitro* sub-cultures [15, 32].

Our results indicate that BR regulates SE induction by activating expression of genes related to embryogenesis and regulating the response to auxin. ABRGs may take part in regulation of gene expression in the process of BR-regulated SE induction. Several SE-related genes were regulated by BR, especially BBM, *PLT2*, *AGL23, LEC2, FUS3, CUC2,* and *CUC3*. Also, exogenously supplemented epiBR resulted in higher expression of those genes. This fact indicates that BRs play a role in the early stage of SEis by activating the expression of these genes. Both *PLT2* and *AIL5* promoted SEis, activating the expression of *LEC1*, *LEC2* and *FUS3* [34, 37]. Expression of *AIL5* and *AIL7* can also activate *CUC1* and *CUC2* in zygotic embryogenesis [39, 40]. The *WUSCHEL (WUS)*-related homeobox (WOX) gene family mediated auxin-triggered SEis in *Arabidopsis* [32]. Our work reported here provided an initial molecular insight in BR regulated SE. We found that the transcriptome of WLI is most similar to that of embryogenic callus with large numbers of genes specifically expressed in both tissues. Genes, especially *BR6ox*, *WOX8*, and *GH3s*, were identified by their connecting edges within the network. Future work showing the interaction of these genes may clarify the unique mode of action of BR in regulating somatic embryogenesis.

Several observations indicate that auxin interacts with BR, and these two hormones operate in a synergistic manner. BR enhances classical auxin growth responses such as hypocotyl elongation [41], lateral root number [42], and gravitropic response [43]. This auxin:BR interplay is also evident at the transcriptional level. Here, we studied whether the auxin was indispensable for BR-induced SE. In this study, epiBR exhibited a synergic, not decisive, function with 2,4-D and 6-BA in SE induction. Similarly, when BR was used solely, rate of SE induction from leaf explants retained no change in coffee [26]. Although auxin is usually required to induce SEis in tissue cultures, only a part of specific tissues respond to auxin induction, entering the pathway of SEis. Auxin induces transcription of many early genes, including *ARF*, *AUX/IAA*, and *SAUR* family members [44]. Auxin-related genes were identified in EC and WLI. Evidences in this study support that BRs activate auxin in sweetgum callus during SE initiation. First, the expression of auxin responsive genes *AUX22* and *SAUR50* increased when the expression of auxin transport gene, *LAX5*, was up-regulated by BR treatment. Second, exogenous BR enhanced *YUC7* expression (Fig. 8), while YUC flavin monooxygenase is a key enzyme in TRP dependent auxin biosynthesis [45].Therefore, BR may not only activate *YUC7*, which encodes auxin biosynthesis enzyme, but also up-regulate auxin responsive genes. Expression of auxin response genes and auxin transport genes could also be activated by BR directly, enhancing plant responses to auxin for SE initiation.

As the conclusion, high correlations were found between the patterns of embryogenicity, endogenous BRs and expression of SEis-related genes with the explants set, which consisted of samples selected by phase changes on their morphological characteristics. Furthermore, stimulating SEis by exogenous BR supplement confirmed the regulatory role of BR during SE initiation and the synergic function of BR with other plant hormones, especially auxin. This research proves that embryogenicity of explants is one of the key factors in initiating SEis, which could be manipulated by *in vitro* conditions, specifically by exogenously supplemented epiBR in hybrid sweetgum. Although this is the first report of induced SEis from leaves in hybrid sweetgum, it is encouraging to induce SEis from mature tissues in other *Liquidambar* species by the treatments of explant with brassinosteroids and/or its precursors before/during SE induction. The research strategy developed in this research could be also applied to other horticulture woody plants.

## Materials and methods

### Plant materials

Mature somatic embryos with two well-developed cotyledons of hybrid sweetgum (*L. styraciflua × L. formosana*) were germinated for two months. The germination medium contained improved Blaydes medium salts [10], plus sucrose (40 g L^−1^) and gellan gum (3 g L^−1^) (phytogel®, Sigma Chemical Co., USA), adjusted to pH 5.7. Cultures were kept at 25 ± 2°C under 16 h light cycles in the culture room of Shen Zhou Lv Peng Agricultural Science & Technology Co. Ltd, Beijing, China. Four types of samples were collected: the normal green leaf (GL); the distal part of white leaf (WLI); the part between the white end and the green end of the same leaf (WLII); and embryogenic callus (EC). Tissue of EC was induced from immature zygotic embryos three years ago approximately, and maintained by regular subcultures once a month. Samples were divided into two parts; one part was preserved in liquid nitrogen for transcriptome analysis, plant hormones determination, and RNA extraction. Another part was inoculated in SE induction medium I in dark at 27 ± 2°C for 30 d and then used for embryogenic capacity evaluation. SE induction medium I contained improved Blaydes medium salts, inositol (0.1 g L^−1^), casein hydrolysate (1 g L^−1^), 2,4-dichlorophenoxyacetic acid (2,4-D, 1 mg L^−1^) and 6-benzylamino-purine (6-BA,0.5 mg L^−1^). Calli used for exogenous treatment were derived from green leaves, which were maintained by regular subcultures on fresh SE induction medium I once per month.

### Exogenous epibrassinolide treatments

Epibrassinolide (epiBR, Sigma Chemical Co., USA) was added to SE induction medium I to achieve the final concentrations of 0, 0.1, 0.5 and 1.0 μM, respectively. Calli (1.0 cm × 1.0 cm) grew in SE induction medium I (20 each) for 30 days before the embryogenic capacity evaluation was made. Three biological replicates were carried out in this experiment.

### Embryogenicity evaluation of explants

Samples were transferred to SE induction medium II, which contained Blaydes medium salts with sucrose (30 g L^−1^) and inositol (0.1 g L^−1^) without plant growth regulators. The culture was kept in dark at 27 ± 2°C for 90 days before SE induction. Embryogenic capacity was judged on the percentage of explants, such as calli, forming SEs to the total explants used. All experiments were repeated three times, and each replicate used at least 60 explants (20 explants/dish).

### Phytohormone analysis

Plant hormones were qualified and quantified according to Wang et al. [46] with the method of Enzyme-Linked Immunosorbent Assay (ELISA). Plant material was homogenized with methanol and centrifuged. The supernatant was then passed through a C18 Sep-Pak column (Waters Corp., Millford, MA, USA), and blown dry with N_2_ at 20°C, and dissolved in phosphate buffered saline. The free plant hormones were quantified by ELISA. Antibodies against the plant hormones were produced by the Center of Crop Chemical Control, China Agricultural University. The average recovery rate of all samples was >90%, which was confirmed with internal standards during extraction and analysis.

### Histological analysis

Leaf tissues were fixed in FAA solution, dehydrated gradually with dimethylbenzene and ethanol. Samples were dyed in Safranine, and discolored in ethanol and dyed in Fast Green before dehydrated with anhydrous ethanol. Finally, samples were hyalinized in dimethylbenzene, embedded in resin (Servicebio, China). Nikon ECLIPSE E100 microscopy and Nikon DS-U3 system was used for image acquisition.

### Analysis of SE induction viability

For SE induction viability analysis, callus from different treatments were stained with 0.01% fluorescein diacetate (FDA) solution diluted in water for 5 min and rinsed with water for 5 times. At least 0.2 g callus from 5 dishes for each treatment were evaluated. Photos were taken using Leica M205FA fluorescence stereo microscope. The experiment was based on three biological replicates, in which one 3.5 × 1.0 cm Petri dishes containing 1.5 mL water were considered as one replicate.

### RNA extraction and quantitative real-time PCR (qRT-PCR)

RNA was extracted using an *EasyPure* Plant RNA Kit (Beijing Transgen Biotech Co. Ltd., Beijing, China). The cDNA was obtained using a *TransScript* One-Step DNA Removal and cDNA Synthesis SuperMix Kit (Beijing Transgen Biotech Co. Ltd., Beijing, China). The qRT-PCR was performed using Trans Start Top Green qPCR SuperMix (Beijing Transgen Biotech Co. Ltd., Beijing, China) on CFX Connect™ Real-Time PCR Detection System (CFX Connect, Bio-Rad, Munich, Germany). The amplification condition for qRT-PCR was under the following program: denaturation at 94°C for 30 s, 40 cycles at 94°C for 5 s, 60°C for 15 s, 72°C for 15 s, with a melt cycle from 65 to 95°C. The relative gene expression in each sample was normalized to *EF1* Ct value and calculated using the 2^−ΔΔCt^ method. The gene-specific primers are listed in Tab. S1. Transcript relative abundance was calculated in triplicate with different cDNAs synthesized from three biological replicates.

### RNA-Seq and bioinformatics

The total RNA was extracted using TRIzol reagent (Invitrogen, Carlsbad, CA, USA). The cDNA libraries were performed using the Illumina HiSeq4000 System (Illumina, San Diego, CA, USA). HISAT2 software was used to compare the clear reads with those of *L. formosana* genome for the efficiency. StringTie was used to assemble the mapped date and analyze, based on uses of FPKM (Fragments Per Kilobase of transcript per Million fragments mapped) as an index to measure the expression levels of transcripts or genes. For analysis of differentially expressed genes, the DESeq2 was performed using Fold Change≧2 and FDR <0.01 as the criteria. Gene functions were annotated based on NR, Swiss-Prot, GO, COG, Pfam, and KEGG. Enrichment analyses were performed using Cluster Profiler. Coexpression networks were constructed using the WGCNA package in R [47]. Eigengene values were calculated for each module and used to test associations with different samples. Networks were visualized using Cytoscape v.3.6.1 [48].

### Statistical analysis

Statistical analyses were performed using IBM SPSS Statistics version 20.0 (SPSS, Chicago, IL, USA). All statistical analysis was based on three independent replicates. Results were presented as means ± the standard error and analyzed using ANOVA, followed by Tuckey test. Differences with *P* < 0.05 were considered statistically significant.

## Supplementary Material

Web_Material_uhab047

## Data Availability

The raw data of the three samples was uploaded to the NCBI under the accession number PRJNA793003. All data supporting this research result can be obtained in the paper and within its Supplementary Materials published online.
