# Supplementary material for: Transcriptomic and physiological analysis identifies a gene network module highly associated with brassinosteroid regulation in hybrid sweetgum tissues differing in the capability of somatic embryogenesis
Source: Hortic Res. 2022 Jan 5;9:uhab047. doi: 10.1093/hr/uhab047 (PMC8788368; doi:10.1093/hr/uhab047)
Supplement: Web_Material_uhab047 [file web_material_uhab047.zip › HORTRES-04190-s01.docx]

**Supplementary information**

**Fig. S1. Endogenous hormone concentrations in the explants.** **A-I**, Concentrations of Brassinosteroid (BR), abscisic acid (ABA), indole-3-acetic acid (IAA), gibberellic acid (GA_3_), gibberellin A4 (GA_4_), zeatin riboside (ZR), dihydrozeatin riboside (dHZR), indolepropionic acid (iPA), and jasmonic acid methyl ester (JA-Me). Mean ± SD, n=3.Values followed by different letters were significantly different at *P*< 0.05.

**
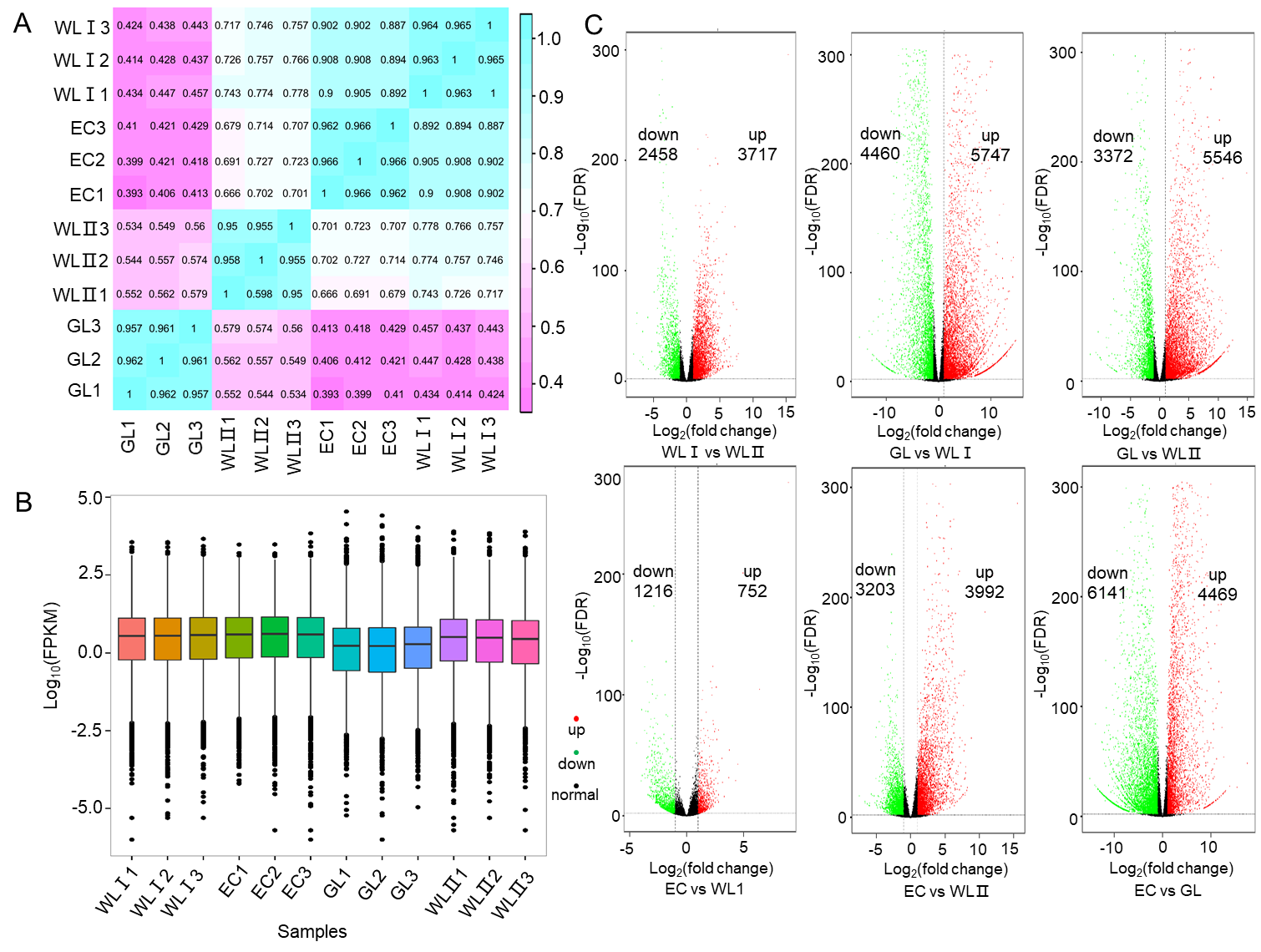
**

**Fig. S2 Gene annotation of full-length isoforms and global gene expression analysis. A**, Pearson correlations between EC, WLⅠ, WLⅡ and GL materials based on expression levels. **B**, Box plot of the FPKM distribution among EC, WLⅠ, WLⅡ and GL materials. **C**, Comparative transcriptome analysis between any two types of explants. The log_2_ (fold change) for each gene is plotted against the -log_10_ (FDR). Significantly differentially expressed genes at an FDR of 5% are highlighted in red for up-regulation and green for down-regulation. EC, embryogenic callus; WLⅠ, white leave part 1; WLⅡ, white leave part 2; and GL, green leave.


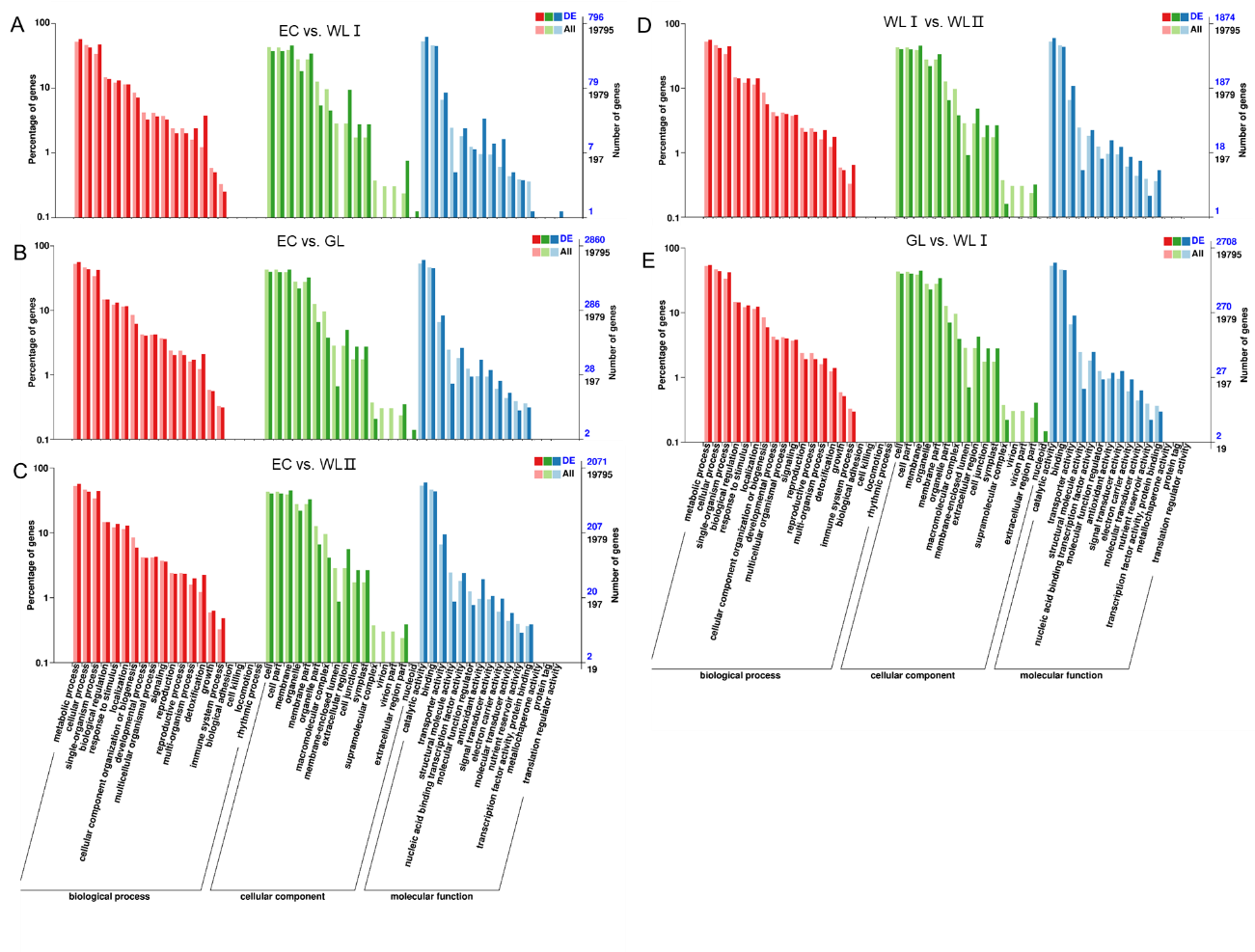


**Fig. S3 Gene Ontology (GO) terms enrichment of ABRGs.** GO terms enrichment of ABRGs in EC vs. WLⅠ (A), EC vs. GL (B), EC vs. WLⅡ (C), WLⅠ vs. WLⅡ (D), and GL vs. WLⅠ (E). GO enrichment was annotated according to biological process, cellular component, and molecular function.
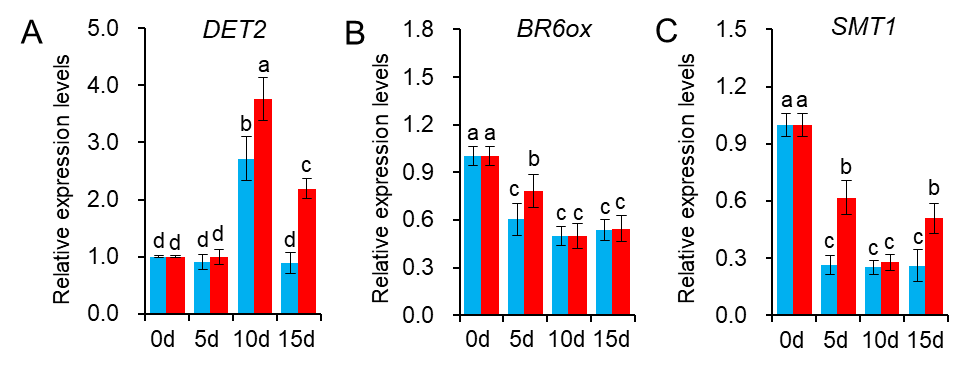


**Fig. S4 Quantitative real-time PCR analysis of genes related to BR and sterols biosynthesis after epiBR treatments.** Mean ± SD, n=3.Values followed by different letters were significantly different at *P*< 0.05.

**Tab. S1 Primer sequences used in qRT-PCR**

| **Number** | **Gene name** | **Gene ID** | **Seq 5'-3'** | |
| --- | --- | --- | --- | --- |
| 1 | *EF1α* | EVM0016344 | F | CCTCTCGGTCGTTTTGCTGT |
|  |  |  | R | GTAGATCTCCCCAAAGGCACTC |
| 2 | *ABI3* | EVM0003173 | F | ATGGAAACCCGAGAAGAACCTA |
|  |  |  | R | ACACCTCCAACAAACACCATTTC |
| 3 | *FUS3* | EVM0000888 | F | AGACTTCATTTTTCTCATGGAACG |
|  |  |  | R | GAATTCCTTCCTTGGACTCGAG |
| 4 | *AIL5* | EVM0005829 | F | TGAAGCACATCTTTGGGACAAC |
|  |  |  | R | GTAGTGGTGGTCGCCCAGTATT |
| 5 | *AIL7* | EVM0008236 | F | TGCAAGTGCCAAACTGAACTTC |
|  |  |  | R | GATTTCTGCTGGTCACTCCTCTG |
| 6 | *AUX22* | EVM0022066 | F | GATCGCATCGAAGCCACAA |
|  |  |  | R | TGAGCTCGGAGTACCCCTTG |
| 7 | *IAA4-L* | EVM0029381 | F | AGAGAGGGAAGGCTACAAGGGT |
|  |  |  | R | ACCGACAGCGAGAGAAAGAGAG |
| 8 | *IAA9-L* | EVM0013806 | F | AACTCTCTTCTGCCCTCGAGAA |
|  |  |  | R | TGCTCTCACTCAGCATCTCCC |
| 9 | *LAX5* | EVM0005994 | F | ACCTGGACAAGAGAACCTGGAC |
|  |  |  | R | CATGGAGGAGGGAAGCAATG |
| 10 | *SAUR50* | EVM0009456 | F | CAACCCCTCTCCCTAAAACTCTC |
|  |  |  | R | TTGCTCATGGAGTGCAGTCTTT |
| 11 | *YUC7* | EVM0001552 | F | CAGAGAAGGTGGTGCCAGAGTT |
|  |  |  | R | TGTGGTTGCAAAGATCAAGAGAG |
| 12 | *LEC2* | Gene_18824 | F | GGACTCATATATAACCAAGGCGG |
|  |  |  | R | ACACACTGGAAATAGTGAACCCAG |
| 13 | *BBM* | EVM0013106 | F | GCCATTTAGCATGCACTACCC |
|  |  |  | R | GACGAGTCCAAGCTCATCAGGT |
| 14 | *PLT2* | EVM0016488 | F | TTGGGAGAATTTTGATCGGTTT |
|  |  |  | R | GCCTGAGATGTATGGAGATGTGG |
| 15 | *WOX2* | EVM0018585 | F | CCGCTTCTCCTGTTTTCCC |
|  |  |  | R | GTCTTCCCCACTTTTTCAGTCC |
| 16 | *SERK1* | EVM0021008 | F | GAAGTTGTCAGAATGCTTGAAGGT |
|  |  |  | R | TCGGTAGAGTCAAGGATCCATTC |
| 17 | *DET2* | EVM0000432 | F | CTCATTATGCCGATTACGATGG |
|  |  |  | R | CTTATACCCACCACCACTTTTCC |
| 18 | *DWF4* | EVM0026210 | F | ACTATTTCTCTCAACTTCCTGAGCC |
|  |  |  | R | CTTTGCTTCATCCTGAGCAGAA |
| 19 | *BR6ox* | EVM0029257 | F | CTGAAACAAGGTCCAAACTTCATG |
|  |  |  | R | ACAGGAAAAGCCTCTTGTCCC |
| 20 | *SMT1* | EVM0011999 | F | GCATATGAGTGGTGCATGACTGA |
|  |  |  | R | CAAACGCTTAATAGCAAATCACAAG |
| 21 | *SMO2* | EVM0028160 | F | GAATATGCTCATCCTGCTGAGATACT |
|  |  |  | R | CACAATGTGCCTCGACTGTCTC |
| 22 | *CYP51* | EVM0017603 | F | AGTACTTATCTGCTGTGTTGGACGA |
|  |  |  | R | TACGTAGCAGCAAAATCAGAGGA |
| 23 | *DWF5* | EVM0022111 | F | CAGTATGAAGAGAATGGGAGAGTAGC |
|  |  |  | R | CTATAAACCTCGGTCATGCGC |
| 24 | *CUC2* | EVM0001055 | F | TGCAGAACTGGCAGAGCTGA |
|  |  |  | R | ACCCTGATATTGTGAAGCATCCT |
| 25 | *CUC3* | EVM0002465 | F | CTCTTCAAGTCCCTCCTCTCACA |
|  |  |  | R | GCCCAACACACTACAATCCATCT |
| 26 | *CLE16* | EVM0021747 | F | CGGCGAGAAAAGTAGGGTTTT |
|  |  |  | R | GTGAAGTGGATTTGGACCAGTGT |
| 27 | *CLE22* | EVM0004164 | F | ATGGCTCTGAGCTGAGTCCTGT |
|  |  |  | R | TTGGACCTGTATGGATTTTCCTC |

**Tab. S2 RNA-Seq information**

| **Sample** | **Read Sum** | **Base sum** | **Total Reads** | **Mapped Reads** | **Uniq Mapped Reads** | **Multiple Mapped Reads** | **Q20 (%)** | **Q30 (%)** | **GC (%)** |
| --- | --- | --- | --- | --- | --- | --- | --- | --- | --- |
| EC_1 | 54,866,339 | 16,333,163,266 | 109732678 | 74375920(67.78%) | 72488660(66.06%) | 1887260(1.72%) | 98.15 | 94.37 | 43.28 |
| EC_2 | 54,190,275 | 16,197,428,926 | 108380550 | 73171039(67.51%) | 71369780(65.85%) | 1801259(1.66%) | 98.11 | 94.34 | 43.24 |
| EC_3 | 53,465,062 | 15,889,856,512 | 106930124 | 70962709(66.36%) | 69214006(64.73%) | 1748703(1.64%) | 97.92 | 93.89 | 43.42 |
| WLI_1 | 53,859,443 | 16,006,154,498 | 107718886 | 74469242(69.13%) | 72620332(67.42%) | 1848910(1.72%) | 98.28 | 94.76 | 43.41 |
| WLI_2 | 52,903,103 | 15,755,604,776 | 105806206 | 72831198(68.83%) | 70998438(67.10%) | 1832760(1.73%) | 98.36 | 94.98 | 43.39 |
| WLI_3 | 52,450,174 | 15,596,509,754 | 104900348 | 73241789(69.82%) | 71405955(68.07%) | 1835834(1.75%) | 98.31 | 94.84 | 43.76 |
| WLII_1 | 52,929,988 | 15,689,933,688 | 105859976 | 73436408(69.37%) | 71801937(67.83%) | 1634471(1.54%) | 98.55 | 95.36 | 43.39 |
| WLII_2 | 59,158,126 | 17,437,875,904 | 118316252 | 82143884(69.43%) | 80203568(67.79%) | 1940316(1.64%) | 98.5 | 95.23 | 43.2 |
| WLII_3 | 57,046,802 | 16,804,321,238 | 114093604 | 79853995(69.99%) | 78135258(68.48%) | 1718737(1.51%) | 98.45 | 95.08 | 43.79 |
| GL_1 | 68,882,406 | 20,247,916,382 | 137764812 | 92607098(67.22%) | 91152847(66.17%) | 1454251(1.06%) | 98.63 | 95.49 | 42.43 |
| GL_2 | 64,365,572 | 18,903,923,204 | 128731144 | 85534213(66.44%) | 84190168(65.40%) | 1344045(1.04%) | 98.56 | 95.36 | 42.74 |
| GL_3 | 57,471,512 | 16,874,911,916 | 114943024 | 77572667(67.49%) | 76488214(66.54%) | 1084453(0.94%) | 98.57 | 95.32 | 42.55 |

**Tab. S3 All DEGs information**

| **DEG Set** | **DEG Number** | **up-regulated** | **down-regulated** | **COG** | **GO** | **KEGG** | **KOG** | **NR** | **Pfam** | **Swiss-Prot** | **eggNOG** |
| --- | --- | --- | --- | --- | --- | --- | --- | --- | --- | --- | --- |
| WLⅠ_vs_WLⅡ | 6175 | 3717 | 2458 | 2679 | 4285 | 2126 | 3005 | 5878 | 5077 | 4818 | 5727 |
| EC_vs_WLⅠ | 1968 | 752 | 1216 | 928 | 1446 | 708 | 940 | 1881 | 1703 | 1648 | 1825 |
| EC_vs_GL | 10610 | 4469 | 6141 | 4632 | 7263 | 3584 | 5276 | 10077 | 8659 | 8042 | 9796 |
| EC_vs_WLⅡ | 7195 | 3992 | 3203 | 3070 | 4904 | 2418 | 3508 | 6793 | 5836 | 5543 | 6610 |
| GL_vs_WLⅠ | 10207 | 5747 | 4460 | 4440 | 7017 | 3453 | 5159 | 9720 | 8347 | 7760 | 9475 |
| GL_vs_WLⅡ | 8918 | 5546 | 3372 | 3898 | 6233 | 3035 | 4400 | 8533 | 7368 | 6882 | 8337 |

**Tab. S4 Gene Ontology (GO) terms enrichment of ABRGs in EC vs. WLⅠ, EC vs. GL, EC vs. WL Ⅱ, WLⅠ vs. WL Ⅱ, and GL vs. WLⅠ.**

| **GO_classify1** | **GO_classify2** | **All** | **WLⅠ vs. WLⅡ** | **EC vs. WLⅠ** | **EC vs. GL** | **EC vs. WLⅡ** | **GL vs. WLⅠ** |
| --- | --- | --- | --- | --- | --- | --- | --- |
| **Total_gene** | | **19795** | **1874** | **796** | **2860** | **2071** | **2708** |
| Cellular component | extracellular region | 561 | 90 | 75 | 142 | 116 | 115 |
|  | cell | 8516 | 755 | 299 | 1125 | 838 | 1087 |
|  | nucleoid | 20 | 1 | 1 | 4 | 2 | 4 |
|  | membrane | 7711 | 853 | 364 | 1229 | 925 | 1210 |
|  | virion | 60 | 0 | 0 | 1 | 0 | 1 |
|  | cell junction | 344 | 50 | 22 | 78 | 55 | 76 |
|  | membrane-enclosed lumen | 563 | 17 | 0 | 19 | 18 | 19 |
|  | macromolecular complex | 1907 | 71 | 36 | 108 | 86 | 107 |
|  | organelle | 5498 | 413 | 145 | 629 | 454 | 614 |
|  | extracellular region part | 47 | 6 | 6 | 10 | 8 | 11 |
|  | organelle part | 2509 | 122 | 43 | 190 | 137 | 189 |
|  | virion part | 60 | 0 | 0 | 1 | 0 | 1 |
|  | membrane part | 5487 | 631 | 273 | 921 | 681 | 917 |
|  | cell part | 8465 | 753 | 298 | 1119 | 834 | 1081 |
|  | symplast | 344 | 50 | 22 | 78 | 55 | 76 |
|  | supramolecular complex | 74 | 3 | 0 | 6 | 1 | 6 |
| Molecular function | transcription factor activity, protein binding | 72 | 10 | 1 | 9 | 8 | 8 |
|  | nucleic acid binding transcription factor activity | 360 | 42 | 19 | 75 | 50 | 67 |
|  | catalytic activity | 10488 | 1119 | 492 | 1724 | 1230 | 1602 |
|  | signal transducer activity | 187 | 23 | 11 | 34 | 22 | 34 |
|  | structural molecule activity | 486 | 10 | 4 | 21 | 18 | 18 |
|  | transporter activity | 1303 | 203 | 68 | 238 | 197 | 244 |
|  | binding | 9167 | 816 | 356 | 1288 | 896 | 1229 |
|  | electron carrier activity | 120 | 16 | 13 | 23 | 20 | 25 |
|  | antioxidant activity | 190 | 29 | 27 | 49 | 40 | 32 |
|  | metallochaperone activity | 3 | 0 | 0 | 0 | 0 | 0 |
|  | protein tag | 3 | 0 | 0 | 0 | 0 | 0 |
|  | translation regulator activity | 2 | 1 | 1 | 1 | 0 | 1 |
|  | nutrient reservoir activity | 78 | 4 | 3 | 8 | 6 | 6 |
|  | molecular transducer activity | 87 | 14 | 4 | 15 | 12 | 17 |
|  | molecular function regulator | 247 | 15 | 9 | 27 | 16 | 25 |
| Biological process | reproduction | 474 | 39 | 16 | 58 | 48 | 52 |
|  | cell killing | 5 | 1 | 0 | 1 | 2 | 1 |
|  | immune system process | 65 | 12 | 2 | 9 | 10 | 8 |
|  | metabolic process | 10394 | 1054 | 459 | 1595 | 1170 | 1483 |
|  | cellular process | 9188 | 785 | 339 | 1251 | 880 | 1184 |
|  | reproductive process | 472 | 39 | 16 | 58 | 48 | 52 |
|  | biological adhesion | 8 | 0 | 0 | 0 | 0 | 0 |
|  | signaling | 734 | 72 | 26 | 103 | 74 | 103 |
|  | multicellular organismal process | 826 | 74 | 29 | 121 | 89 | 108 |
|  | developmental process | 836 | 69 | 26 | 116 | 85 | 103 |
|  | growth | 117 | 10 | 4 | 16 | 13 | 14 |
|  | locomotion | 5 | 0 | 0 | 0 | 0 | 0 |
|  | single-organism process | 6688 | 830 | 375 | 1210 | 917 | 1140 |
|  | rhythmic process | 3 | 0 | 0 | 0 | 0 | 0 |
|  | response to stimulus | 2404 | 264 | 105 | 379 | 283 | 350 |
|  | localization | 2261 | 267 | 91 | 332 | 269 | 335 |
|  | multi-organism process | 316 | 42 | 19 | 49 | 41 | 53 |
|  | biological regulation | 2908 | 267 | 110 | 421 | 301 | 392 |
|  | cellular component organization or biogenesis | 1682 | 105 | 57 | 178 | 123 | 161 |
|  | detoxification | 244 | 33 | 30 | 60 | 47 | 38 |

**Tab. S5 Genes of ABRGs involved in auxin-related and embryogenesis-related pathways in explants of EC, WLⅠ, WLⅡ and GL of hybrid sweetgum.**

| **Pathway** | **Gene name** | **Sweetgum gene ID** | **Araabidopsis gene ID** | **NR_annotation** |
| --- | --- | --- | --- | --- |
| auxin-related genes | *ARF2B-L* | EVM0000336 | AT5G62000 | auxin response factor 2B-like isoform X1 [Prunus avium] |
|  | *ARF5* | EVM0026553 | AT1G19850 | PREDICTED: auxin response factor 5 [Vitis vinifera] |
|  | *ARF6* | EVM0001306 | AT5G37020 | auxin response factor 6 [Morus notabilis] |
|  | *ARF8* | EVM0008817 | AT5G37020 | ThiJ/PfpI [Corchorus olitorius] |
|  | *ARF9* | EVM0011769 | AT4G23980 | PREDICTED: auxin response factor 9 [Vitis vinifera] |
|  | *ARF18* | EVM0001543 | AT2G28350 | auxin response factor 18 [Quercus suber] |
|  | *AUX2* | EVM0013040 | AT5G01240 | auxin transporter-like protein 2 [Jatropha curcas] |
|  | *AUX22* | EVM0022066 | AT3G15540 | PREDICTED: auxin-induced protein AUX22 [Ricinus communis] |
|  | *AUX6B-L* | EVM0029187 | AT5G10990 | PREDICTED: auxin-induced protein 6B-like [Vitis vinifera] |
|  | *IAA1* | EVM0003074 | AT1G04250 | PREDICTED: auxin-responsive protein IAA1 [Nelumbo nucifera] |
|  | *IAA13-L* | EVM0027798 | AT2G33310 | PREDICTED: auxin-responsive protein IAA13-like isoform X2 [Juglans regia] |
|  | *IAA1-L* | EVM0018879 | AT3G04730 | PREDICTED: auxin-responsive protein IAA1-like [Nelumbo nucifera] |
|  | *IAA26* | EVM0001755 | AT3G16500 | PREDICTED: auxin-responsive protein IAA26 [Tarenaya hassleriana] |
|  | *IAA33* | EVM0029406 | AT5G57420 | auxin early response protein AUX/IAA33 [Camellia sinensis] |
|  | *IAA4-L* | EVM0000528 | AT5G43700 | auxin-responsive protein IAA4-like [Quercus suber] |
|  | *IAA4-L* | EVM0013779 | AT5G43700 | PREDICTED: auxin-responsive protein IAA4-like [Nelumbo nucifera] |
|  | *IAA4-L* | EVM0029381 | AT5G43700 | auxin-responsive protein IAA4-like [Quercus suber] |
|  | *IAA9* | EVM0013754 | AT5G65670 | auxin early response protein AUX/IAA9 [Camellia sinensis] |
|  | *IAA9-L* | EVM0013806 | AT5G65670 | auxin-responsive protein IAA9-like [Hevea brasiliensis] |
|  | *SAUR21* | EVM0026314 | AT4G34810 | PREDICTED: auxin-responsive protein SAUR21 isoform X2 [Vitis vinifera] |
|  | *SAUR36* | EVM0010505 | AT3G60690 | Small auxin-up RNA [Trema orientalis] |
|  | *SAUR41-L* | EVM0009456 | AT1G72430 | PREDICTED: auxin-responsive protein SAUR41-like [Juglans regia] |
|  | *SAUR50* | EVM0005939 | AT1G75590 | auxin-induced protein 6B [Cucurbita moschata] |
|  | *DRM1* | EVM0004575 | AT1G28330 | Dormancyauxin associated [Parasponia andersonii] |
|  | *GH3.17* | EVM0027525 | AT1G28130 | indole-3-acetic acid-amido synthetase GH3.17-like [Quercus suber] |
|  | *GH3.3* | EVM0013113 | AT2G14960 | GH3 auxin-responsive promoter [Corchorus olitorius] |
|  | *GH3.5* | EVM0026336 | AT4G27260 | Auxin-responsive GH3 family protein [Theobroma cacao] |
|  | *GH3.6* | EVM0018937 | AT5G54510 | PREDICTED: indole-3-acetic acid-amido synthetase GH3.6 [Vitis vinifera] |
|  | *VAB* | EVM0022644 | AT5G43870 | VAN3-binding protein [Quercus suber] |
|  | *PID* | EVM0011776 | AT2G34650 | PREDICTED: protein kinase PINOID [Vitis vinifera] |
|  | *LAX3* | EVM0025634 | AT1G77690 | auxin transporter-like protein 3 [Hevea brasiliensis] |
|  | *LAX5* | EVM0005994 | AT2G21050 | PREDICTED: auxin transporter-like protein 5 [Juglans regia] |
|  | *PIN6* | EVM0001664 | AT1G77110 | PREDICTED: auxin efflux carrier component 6 [Vitis vinifera] |
|  | *ABCB1* | EVM0017139 | AT2G36910 | PREDICTED: ABC transporter B family member 1 [Juglans regia] |
|  | *ABCB1* | newGene_8946 | AT2G36910 | PREDICTED: ABC transporter B family member 1-like [Populus euphratica] |
|  | *CHS* | EVM0027794 | AT5G13930 | Chal_sti_synt_N domain-containing protein/Chal_sti_synt_C domain-containing protein [Cephalotus follicularis] |
|  | *CYP711A1* | EVM0006563 | AT2G26170 | cytochrome P450 711A1-like isoform X1 [Prunus avium] |
|  | *NPY1* | EVM0005838 | AT4G31820 | PREDICTED: BTB/POZ domain-containing protein NPY1 [Vitis vinifera] |
|  | *WAT1* | EVM0002799 | AT1G75500 | protein WALLS ARE THIN 1 [Carica papaya] |
|  | *WAT1* | EVM0027302 | AT1G75500 | PREDICTED: protein WALLS ARE THIN 1-like [Nelumbo nucifera] |
|  | *DAO* | EVM0000033 | AT1G14130 | PREDICTED: 2-oxoglutarate-dependent dioxygenase DAO [Vitis vinifera] |
|  | *HST* | EVM0021380 | AT5G48930 | Transferase [Macleaya cordata] |
|  | *YUC5* | EVM0010671 | AT4G28720 | probable indole-3-pyruvate monooxygenase YUCCA5 [Jatropha curcas] |
|  | *YUC7* | EVM0001552 | AT1G04610 | PREDICTED: probable indole-3-pyruvate monooxygenase YUCCA7 [Ziziphus jujuba] |
| Embryogenesis -related genes | *BBM* | EVM0013106 | AT5G17430 | PREDICTED: AP2-like ethylene-responsive transcription factor BBM1 [Nelumbo nucifera] |
|  | *PLT2* | EVM0016488 | AT1G51190 | AP2-like ethylene-responsive transcription factor PLT2 isoform X1 [Hevea brasiliensis] |
|  | *AGL15* | EVM0001986 | AT5G13790 | PREDICTED: agamous-like MADS-box protein AGL15 isoform X1 [Ricinus communis] |
|  | *AGL23* | EVM0028139 | AT2G22630 | PREDICTED: MADS-box transcription factor 23 [Theobroma cacao] |
|  | *AIL5* | EVM0005829 | AT5G57390 | PREDICTED: AP2-like ethylene-responsive transcription factor AIL5 [Nelumbo nucifera] |
|  | *AIL7* | EVM0008236 | AT5G57390 | AP2-like ethylene-responsive transcription factor AIL7 isoform X1 [Ananas comosus] |
|  | *WOX11* | EVM0014412 | AT3G03660 | PREDICTED: WUSCHEL-related homeobox 11 isoform X4 [Theobroma cacao] |
|  | *WOX8* | EVM0003362 | AT4G35550 | PREDICTED: WUSCHEL-related homeobox 8 [Vitis vinifera] |
|  | *CLE22* | EVM0004164 | AT2G01505 | hypothetical protein CFP56_43427 [Quercus suber] |
|  | *CLE16* | EVM0021747 | AT2G01505 | PREDICTED: CLAVATA3/ESR (CLE)-related protein 16 [Juglans regia] |
|  | *CUC2* | EVM0001055 | AT2G24430 | NAC domain-containing protein 92 isoform X3 [Manihot esculenta] |
|  | *CUC3* | EVM0002465 | AT1G76420 | NAC domain transcriptional regulator superfamily protein [Theobroma cacao] |
